# What have we learned from a decade of patient involvement in OMERACT and its effect on trial outcome assessments?

**DOI:** 10.1186/1745-6215-12-S1-A80

**Published:** 2011-12-13

**Authors:** John R Kirwan, Maarten de Wit

**Affiliations:** 1 School of Clinical Sciences, University of Bristol, Bristol UK and Academic Rheumatology Unit, Bristol Royal Infirmary, Bristol, UK; 2 Department of Medical Humanities, VU Medical Centre, Amsterdam, The Netherlands

## Background

Since 2002 people with rheumatic diseases have participated in the bi-annual 5-day international conference and workshop on Outcome Measures in Rheumatology (OMERACT), which uses evidence-based consensus to determine core outcome measures for clinical trials of rheumatological conditions. Patients contribute their perspective and collaborate as equal partners in all conference sessions. In all, 46 patients with 7 different conditions from 12 countries have participated in OMERACT. With the help of professionals they have organized themselves and developed various support mechanisms.

## Methods

The authors have called on their personal experiences of OMERACT, a review of OMERACT publications and discussions with long-term OMERACT participants in setting out results and drawing conclusions.

## Results

The effect of patient involvement has been threefold:

First, the OMERACT research agenda has been enriched by the identification of new domains that are relevant from a patient perspective. The most important example has been the increased efforts to gain more knowledge about the nature and impact of fatigue in rheumatoid arthritis. Fatigue has been added to the rheumatoid arthritis core-set and is now widely measured as an important outcome in clinical trials.

Second, involvement of patients in the structure of the meetings has given insights into barriers to patient participation and to ways of facilitating the incorporation of the patient perspective in outcome research. Before the start of the conference patient participants need appropriate information about the objective and program of the conference and the expected contributions. They need training, support and encouragement to be able to fully collaborate in all parts of the program. And challenges like the patient-doctor relationship, ethics, confidentiality and communication need to be addressed.

Third, OMERACT has set a standard for patient involvement in health outcome research that has led to new local, national and international, initiatives.

## Conclusions

Building the involvement of patients directly into the OMERACT meeting program has significantly contributed to the success of this biannual conference. It has broadened the research agenda of OMERACT, it has had a major spin off on patient involvement in research projects on a national level and it has set a new standard for patient involvement in health outcome research. However, there remains the challenge of fully developing patient participation in the research working groups that meet between the conferences.

